# Nanointerfaces: Concepts and Strategies for Optical and X-ray Spectroscopic Characterization

**DOI:** 10.1021/acsphyschemau.2c00058

**Published:** 2023-02-09

**Authors:** Tristan Petit, Mailis Lounasvuori, Arsène Chemin, Peer Bärmann

**Affiliations:** Nanoscale Solid−Liquid Interfaces, Helmholtz-Zentrum Berlin für Materialien und Energie GmbH, Albert-Einstein Strasse 15, 12489 Berlin, Germany

**Keywords:** nanomaterials, nanoparticles, solid−liquid interface, X-ray spectroscopy, infrared spectroscopy

## Abstract

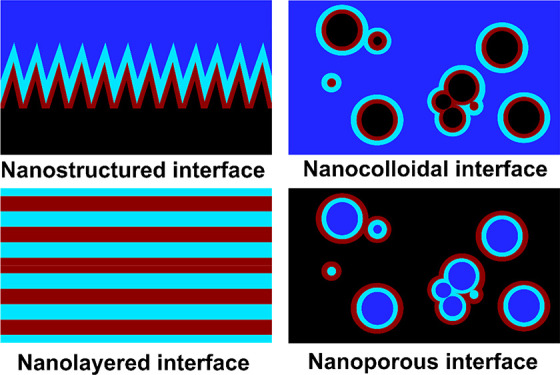

Interfaces at the nanoscale, also called nanointerfaces, play a fundamental role in physics and chemistry. Probing the chemical and electronic environment at nanointerfaces is essential in order to elucidate chemical processes relevant for applications in a variety of fields. Many spectroscopic techniques have been applied for this purpose, although some approaches are more appropriate than others depending on the type of the nanointerface and the physical properties of the different phases. In this Perspective, we introduce the major concepts to be considered when characterizing nanointerfaces. In particular, the interplay between the characteristic length of the nanointerfaces, and the probing and information depths of different spectroscopy techniques is discussed. Differences between nano- and bulk interfaces are explained and illustrated with chosen examples from optical and X-ray spectroscopies, focusing on solid–liquid nanointerfaces. We hope that this Perspective will help to prepare spectroscopic characterization of nanointerfaces and stimulate interest in the development of new spectroscopic techniques adapted to the nanointerfaces.

## 1 Introduction

Many fundamental chemical reactions and adsorption processes governing applications in energy storage and energy conversion, catalysis, and biology, among others, are taking place at interfaces.^1^ The ever-increasing interest in nanomaterials is largely motivated by their large surface-to-volume ratio, which dramatically enhances interfacial processes. Increasing surface area through nanostructuration has become a successful strategy to improve the chemical and catalytic reactivity^2,3^ or energy storage properties^4^ of nanomaterials. Nevertheless, properties which are not simply related to a larger surface area also appear, such as (quantum) confinement effects or more reactive edge/defect states. Over the last years, the controlled synthesis of model nanomaterials with uniform structure and interfacial properties has enabled a better molecular understanding of interfacial effects on nanomaterials.^5^ The characterization of interfaces involving nanomaterials, referred to as nanointerfaces in the following, is therefore gaining momentum.

A variety of spectroscopic techniques have already been applied to provide optical, chemical, or electronic information on nanointerfaces. Depending on the excitation wavelength, the probing depth of the techniques may affect the information depth, from where spectral information is recorded. As a result, spectroscopic techniques with a short information depth (usually <5–10 nm) are often referred to as “surface-” or “interface-sensitive” while techniques with longer information depths (>100 nm) are labeled as “bulk-sensitive”. This classification may become misleading when considering nanointerfaces with characteristic lengths of the same order of magnitude as the probing and information depths. Indeed, for nanomaterials, some “bulk” properties from their core region may be accessed through “surface” sensitive techniques,^6^ while “interfacial” properties associated with their surface chemistry may also be probed by classically “bulk-sensitive” techniques.^7^ It is therefore of high relevance to clarify the interplay between the characteristic length of the nanointerfaces and the probing and information depths of spectroscopic techniques used for their characterization.

In this Perspective, we would like to introduce the main concepts to be considered for the characterization of interfacial phenomena involving nanomaterials. First, a definition and taxonomy of nanointerfaces is proposed. The relevance of nanointerfaces is highlighted, taking the specific field of electrochemical energy storage as an example. Then, the concepts of probing and information depths are explained, which are of uttermost importance for spectroscopy at nanointerfaces. Finally, these concepts are illustrated by selected examples from the literature based on infrared and X-ray spectroscopies. We do not attempt to give an exhaustive review of spectroscopic techniques developed for *in situ*/*operando* characterization of interfaces, which can be found elsewhere for more specific fields. While we mostly focus on solid–water nanointerfaces, the concepts introduced here hold for other types of nanointerfaces.

## 2 General Concepts

### 2.1 Definition of Nanointerfaces

The term nanointerface has been regularly used in the nanomaterials’ community over the last years,^8,9^ but a clear definition has been lacking so far. By analogy with the EU definition of nanomaterials,^10^ we propose to define a nanointerface as the boundary between two phases, with at least one of the phases having one or more characteristic dimensions in the size range of 1–100 nm. The characteristic dimension, or length, is an external dimension that defines the scale of the phase and can be, for example, the diameter of a nanoparticle or the length of a nanowire. Each phase has three characteristic lengths for the three spatial coordinates, which defines its dimensionality: 0D when the three characteristic lengths are nanometric (nanoparticles, quantum dots, or nanobubbles), 1D when two are nanometric (nanowire, nanotube, or nanochannels), and 2D when only one dimension is nanometric (nanoflakes, nanoplatelets, or nanoslit). Note that 2D materials with a characteristic length smaller than 1 nm due to mono- or few atom-thin 2D planar arrangements are usually still considered as nanomaterials.

**Figure 1:**
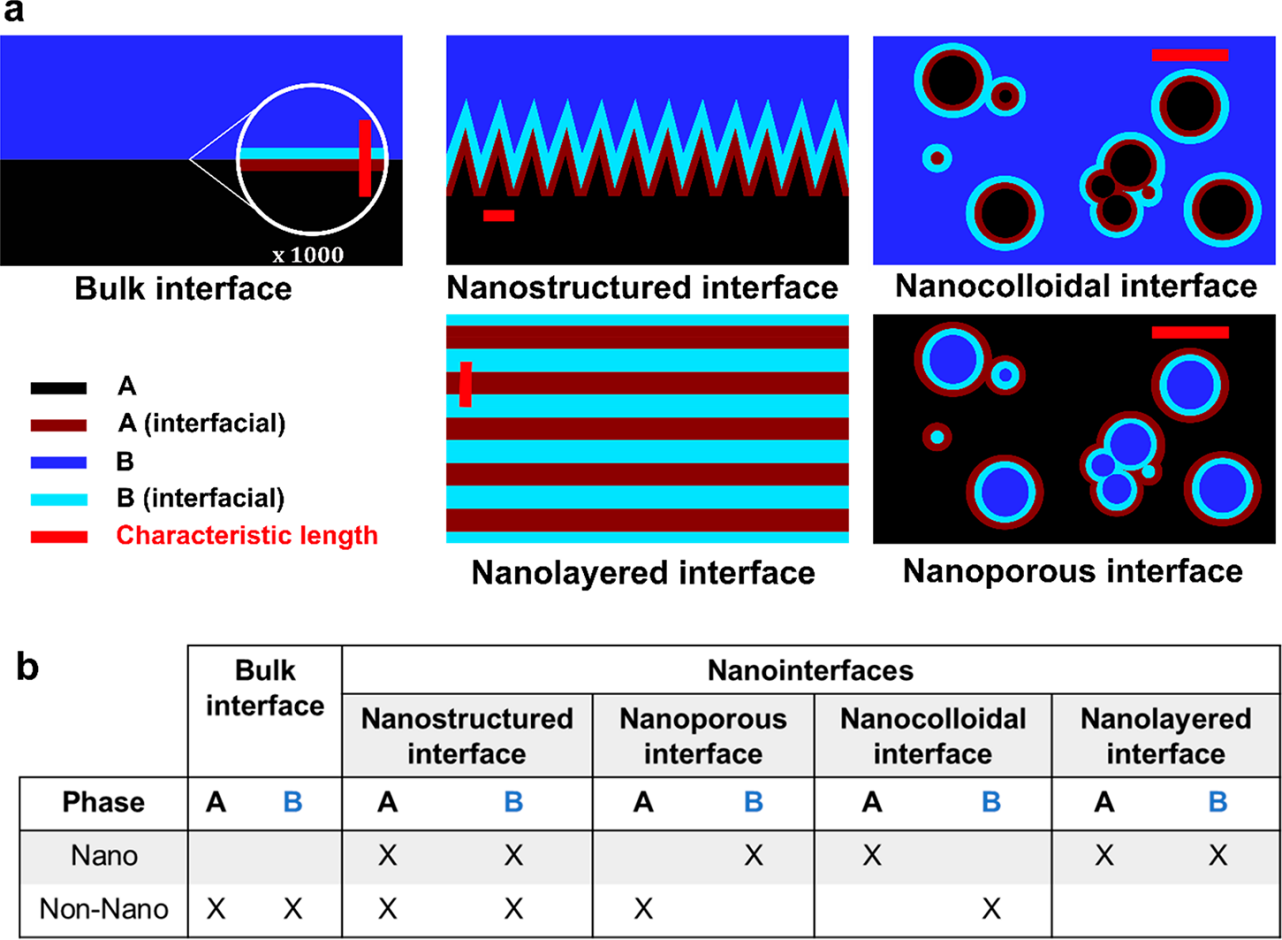
Schematic representation of the four classes of nanointerfaces and bulk interface between a solid (A) and a liquid (B) phase. A characteristic length in the nanoscale range is highlighted in red for all interfaces. A magnified view of the bulk interface is shown to highlight the different scaling factor compared to nanointerfaces. The volume affected by interfacial phenomena, corresponding to the interfacial region, or interphase, is presented in a different color. (b) Classification of nanointerfaces based on the characteristic lengths (nano or non-nano) of the phases A and B.

According to this definition, a taxonomy of nanointerfaces can be derived. Four main classes of nanointerfaces between two media, A and B, are possible based on whether A and/or B have their respective characteristic lengths at the nanoscale, as illustrated in Figure 1a. Further subclasses can be defined based on the dimensionality of each phase if required. Note that both phases involved in nanointerfaces do not necessarily need to have a “nano” characteristic length. When the characteristic length is larger than 100 nm, it will be termed “non-nano”. We will show later that the presence of a “non-nano” phase will have consequences on the spectroscopic signature of the nanointerface; therefore, the distinction between the different nanointerfaces based on their characteristic lengths, as shown in Figure 1b, is required. An interface between two phases which do not have any nanoscale dimension constitutes a bulk interface. This definition is valid for any type of phases A and B (solid, liquid, or gas), but we concentrate here on solid–liquid nanointerfaces, where A is a solid phase and B a liquid phase. The following nanointerfaces are possible:

- *Nanostructured interface*: A solid material with macroscale dimensions and a nanostructured surface form a nanostructured interface when exposed to a liquid phase. In this case, both phases have non-nano and nanoscale components.
- *Nanocolloidal interface*: A colloidal dispersion of a solid nanomaterial in a liquid phase leads to a nanocolloidal interface. Large interfacial areas are formed when the size of the nanomaterial shrinks down and the nanomaterial concentration is high.
- *Nanoporous interface*: A porous solid material which pores are filled with a liquid phase leads to a nanointerface when the pores have nanoscale dimensions.
- *Nanolayered interface*: When both phases have nanoscale dimensions only, they form an ideal nanointerface. This is for example the case for layered 2D materials with a liquid phase confined in the interlayer spacing constituting nanoslits as shown in Figure 1a.

At the interface, both the solid and the liquid phases will have an interfacial volume with properties that differ from the bulk volume. This interfacial volume, also known as “interphase”, usually spans only a few nanometers on both sides of the nanointerface. Villevieille recently summarized the difference between the interface and interphase.^11^Figure 1a highlights the interfacial components of both phases A and B, which together constitute the interphase. For the solid phase, this is related to the volume affected, for example, by surface termination, surface stress, or interfacial band bending. For the liquid phase, solvent restructuring or electrical double layer (EDL) formation can occur in this volume. At nanointerfaces, the interfacial region may constitute a significant portion of the total volume as the characteristic length of the investigated phases approaches the nanometer scale. In the ultimate case of nanolayered interfaces with few atom-thick 2D materials intercalated with an electrolyte, essentially all atoms are involved in interfacial phenomena, and no “core” or “bulk” of nanomaterial and electrolyte can be defined anymore. The importance of nanointerfaces in the context of electrochemical energy storage is highlighted in the following section.

### 2.2 Nanointerfaces for Electrochemical Energy Storage

**Figure 2:**
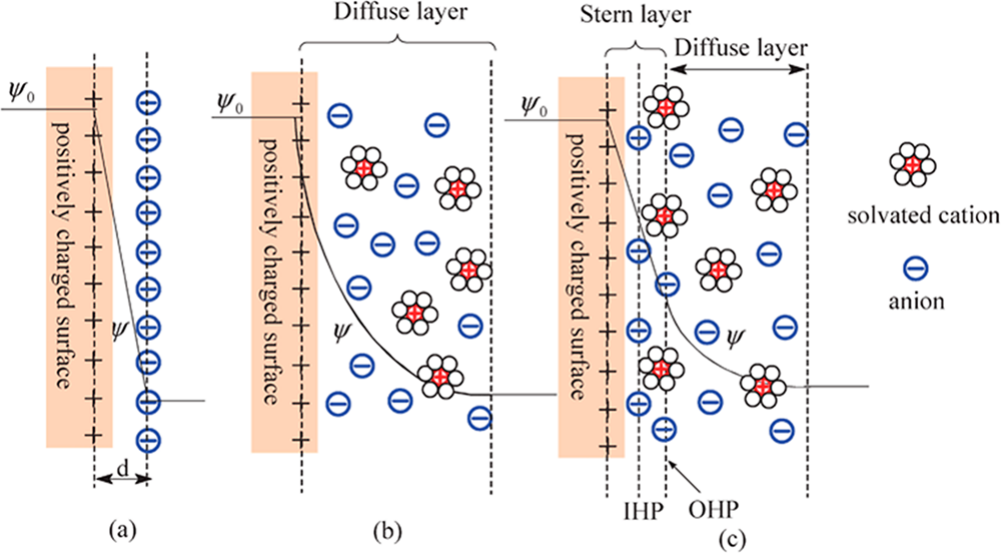
Historical advancement of the “classical” EDL theory, showing the theories developed by Helmholtz (a), Guoy and Chapman (b), and Stern (c). Reproduced with permission from ref (17). Copyright 2009 Royal Society of Chemistry.

The electrode|electrolyte interphase has been under investigation for more than 170 years and was first described by Helmholtz, who investigated colloid particles and envisioned the electrical double layer (EDL) to consist of atomistic layers of opposite charge with a linearly decreasing potential (Figure 2a).^12^ Precisely 60 years later, this revolutionary but simplistic model was improved by Guoy and Chapman independently of each other by breaking the rigid order of the Helmholtz double layer under the consideration of thermal motion of the cationic and anionic species in the electrolyte following the Poisson–Boltzmann theory and thereby defining the EDL as a diffuse layer (Figure 2b).^13,14^ Furthermore, to address the question of the spatial distribution of the EDL into the electrolyte, Stern combined both aforementioned theories by taking the adsorption of ions into account and therefore envisioning the EDL to consist of two different layers, an inner layer (compact layer or Stern layer) and a diffuse layer as defined by Guoy and Chapman.^15^ The compact layer was further split into the inner (IHP) and outer Helmholtz layer (OHP) by Graham in 1947 to account for the specific ionic species, which is today considered as the classical theory of EDL (Figure 2c).^16−18^

Although there are many parameters affecting the EDL (e.g., solvent, salt, salt concentration, electrode, material, functional groups), none have had an impact as fundamental as the presence of nanopores or nanoconfinement for application in electrochemical storage systems, more precisely supercapacitors, in the past few decades. Briefly, supercapacitors store energy through the formation of an EDL, which can be considered as a capacitor, for which the capacitance *C* is defined as
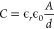
with ϵ_*r*_ and ϵ_0_ the relative and vacuum permittivities, *A* the interfacial area, and *d* the distance between the opposite charges. Nanomaterials can reach much higher capacitance values than conventional capacitors due to the atomic scale of the EDL (*d*) and the large surface area (*A*, e.g. 3000 m^2^ g^−1^ for activated carbon).^17,19−21^

**Figure 3:**
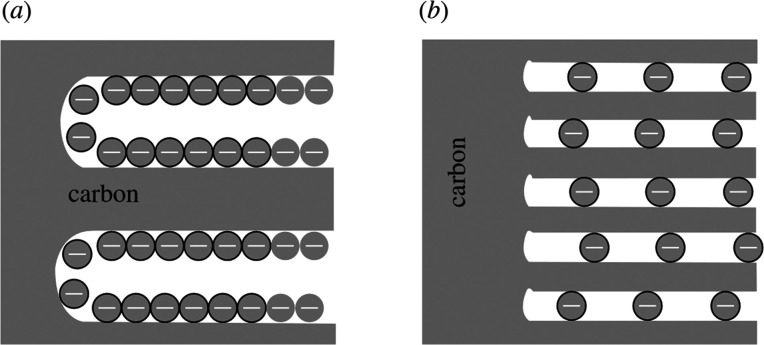
Comparison of the double layer in the “classical“ sense (a) and in a nanoconfined space (b). Reproduced with permission from ref (21). Copyright 2010 Royal Society.

When synthesizing large surface nanomaterials, the pore distribution can be quite broad and ranges from nanopores (<2 nm) to mesopores (2–50 nm) and macropores (>50 nm).^22,23^ Traditionally, nanopores were believed to hamper the electrochemical performance due to a limited ion accessibility that would hinder the EDL formation.^17,24,25^ Although it was shown as early as 1977 that EDL formation is possible in nanopores as small as 0.377 nm through desolvation of the cationic species,^26^ it took until 2006 to prove that pores smaller than 1 nm can lead to increased capacitance values.^25,27^ These groundbreaking results are based on the unimodal pore design of a carbide-derived-carbon structure, which challenged the general understanding of the contribution of pores smaller than the solvated ions to the overall capacitance of supercapacitors as the highest capacitances are achieved with pore sizes comparable to the ion size.^21,28^ This phenomenon cannot be explained by employing “classical” EDL models from which the adsorption of the ionic species at the pore walls would be expected (Figure 3a). But, since the confined space is insufficient to give room for both the Stern and the diffuse layer, the ionic species is stacked inside the pores (Figure 3b).^21^ Such confinement effects provide new opportunities for electrochemical energy storage, but the molecular understanding of charging processes in such an environment is still at its infancy. It has been recently proposed that a continuous transition between fully solvated and fully desolvated ions occurs.^29^*Operando* characterization of ion solvation shells is however needed to provide a physicochemical understanding of the observed electrochemical behavior in nanoconfinement.

This short description of nanointerfaces for electrochemical storage systems emphasizes the importance of having advanced theoretical and experimental methods to unravel the unique physical and chemical properties of nanointerfaces. *In situ/operando* spectroscopy is essential for this purpose, and we discuss in the following section important considerations for probing the EDL and electrochemical reactions at nanointerfaces.

### 2.3 Probing versus Information Volume

**Figure 4:**
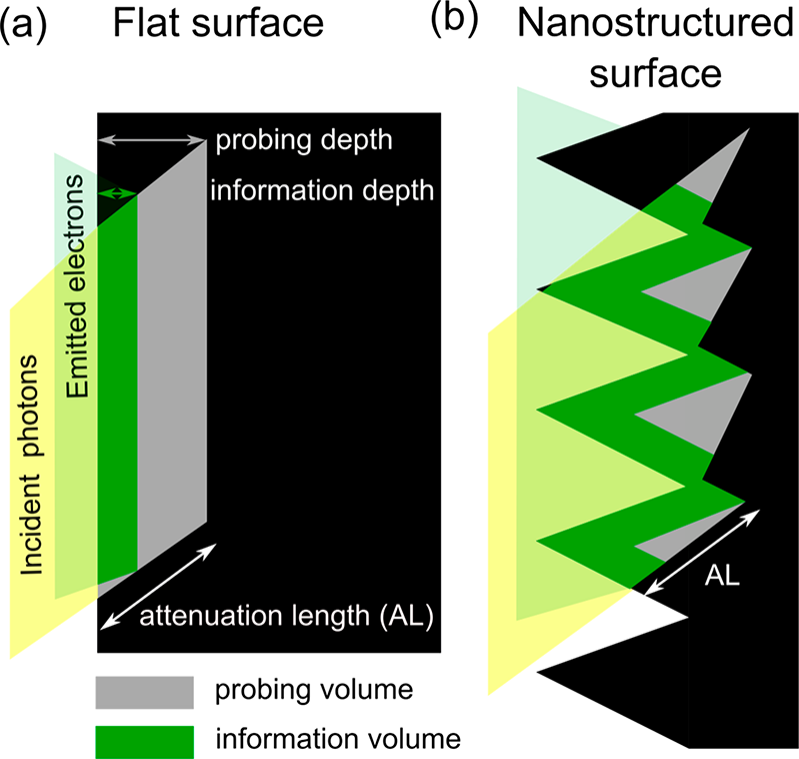
Schematic representation of probing (grey) and information (green) depths and volumes on flat (a) and nanostructured (b) surfaces. An example is given for a photon-in electron-out spectroscopy technique such as XPS. The characteristic length of the nanostructures is of the same order of the attenuation length (AL) of the incident photons in this material.

Spectroscopy is based on the interaction between an electromagnetic wave and the sample to be characterized. Several physical terms such as inelastic mean free path, attenuation length, or mean escape depth are used to refer to different depths or volumes involved in light–matter interactions.^30^ In the context of nanointerfaces, probing depth (volume) and information depth (volume) are particularly relevant. The probing depth relates to the depth of the sample, normal to the surface, which is exposed to the electromagnetic wave and depends on the excitation energy as well as the sample composition. On flat surfaces, the probing depth is calculated from the attenuation length of the incident photons at a given energy and the angle of incidence of the excitation beam.^31^ The attenuation length depends on the material and the excitation energy. On nanomaterials with a more complex morphology, considering a probing volume is more appropriate than a probing depth because the presence of curvature effects or rough morphology will affect the angle of incidence of X-rays and complicate the definition of a normal plane to the surface (Figure 4). Instead of considering nonuniform probing depths, a probing volume can be defined as the overall volume exposed to the electromagnetic waves.

The information depth (volume), on the other hand, refers to the depth (volume) from where a specific percentage (typically 95% or 99%) of the spectral information is recorded. The information volume depends on the detection technique that is used to record spectral information. It can be equal or smaller than the probing volume. A typical example of a spectroscopy technique with different probing and information depths is XPS, which is a photon-in electron-out technique, as shown in Figure 4. The probing X-rays have a longer attenuation length than the detected photoelectrons. One can only access the information on a small part of the excited material from which the photoelectron can escape. In the remaining excited volume, the electrons are absorbed by the sample and are not detected. The information volume is then smaller than the probing volume. In the following, the probing and information volumes will be compared to the characteristic volumes of the nanointerface.

### 2.4 Strategies for Spectroscopic Characterization of Nanointerfaces

Now that the main concepts of nanointerfaces, probing, and information volumes have been introduced, the various strategies to detect spectral information from nanointerfaces can be overviewed:

- *Matching the probing volume and the volume of the nanointerface (S1):* Ideally, only the volume of the nanointerface should be probed. When both phases A and B of the nanointerface have only nanoscale characteristic lengths (such as nanolayered interfaces), even spectroscopy techniques with large probing volumes will probe only interfacial signal. When both macro- and nanoscale components are included in the nanointerface (see Figure 1), the volume of the nanoscale component relative to the bulk component must be increased. Ideally, the bulk phase should be completely suppressed to allow probing only the interfacial region. For example, in a colloidal dispersion, reducing the nanoparticle size will reduce the spectral signal from the nanoparticle core, and increasing its concentration will reduce the signal from the bulk electrolyte. Comparing spectra with different nanoparticle sizes and/or concentrations can often help to resolve the interfacial component in the spectroscopic data. In this case, a spectroscopy technique with a probing and detection volume much larger than the characteristic volume of the nanointerface will still provide a significant portion of signal coming from the nanointerface. Although counterintuitive at first, this explains why spectroscopy methods which are considered as bulk-sensitive on classical interfaces such as IR spectroscopy become interface-sensitive when applied to nanointerfaces.
- *Matching the information depth to the characteristic length of the nanointerface (S2):* if the probing depth is much larger than the characteristic length of the nanointerface, the information depth can be reduced. In the previous example, XPS was shown to be highly surface sensitive because of the detection of electrons having small attenuation length and thereby ensuring a small information depth even though a large volume is excited by X-rays. The bulk component of the probed materials is hence not contributing to the spectral signature. If one would instead detect the photons emitted as secondary process following the X-ray absorption, the bulk component of the probed materials would contribute more to the spectral signature due to the longer attenuation length of X-ray photons.
- *Ensuring selective sensitivity to phase A or B (S3):* A spectroscopic technique with high selectivity to either the phase A or B may allow the removal of bulk contributions, thereby increasing sensitivity to the nanointerface. When phases A and B are constituted of different elements, the element sensitivity of X-ray spectroscopy can be used to probe both phases separately. This approach is particularly successful when either A or B has only a nanoscale component (colloidal dispersion or nanoporous interface). If not, probing and information volumes also have to be optimized using the two previous strategies.
- *Ensuring selective sensitivity to the interface (S4):* Local changes of optical and/or electronic properties in the interfacial region can also be used to probe selectively interfacial signal. High selectivity may be achieved by specific selection rules for optical spectroscopy. A typical example is the change of the refractive index at the interface, which ensures a high selectivity to interfacial signal with Sum Frequency Generation (SFG) techniques.^32,33^

In general, the best strategy will depend on the type of nanointerfaces (Figure 1) as well as the type of spectral information of interest. In the context of *in situ/operando* characterization of nanointerfaces for electrochemical energy conversion and storage, further constraints need to be considered. Following electrochemical processes at nanointerfaces during operating conditions implies the use of electrochemical cells as close as possible to real devices. In general, such cells are based on a 2- or 3-electrode system with a window transparent to the absorbed and emitted photons (or electrons).^34^ The active nanointerface is therefore buried in a complex cell design, which requires spectroscopic techniques with relatively long probing depths.^35^ On the other hand, maintaining an information volume sensitive to interfacial signal is also required. Having these constraints in mind is necessary to carefully design experimental schemes relevant for practical *in situ*/*operando* electrochemical characterizations. The different strategies mentioned above will be illustrated with specific examples of nanointerfaces characterized by optical and X-ray spectroscopies in the next sections.

## 3 Optical Spectroscopic Characterization of Nanointerfaces

### 3.1 Probing and Information Depths for Optical Spectroscopies

In optical spectroscopy, the absorption or the reflectance of a sample is measured following its excitation by an electromagnetic wave. Depending on the wavelength, the incident photons have different energies and excite different transitions. IR spectroscopy probes the vibrational modes of chemical bonds and is particularly sensitive to the surface termination of a material and its interaction with a solvent, for instance. Increasing the energy, the UV/visible range can probe the transition between two electronic states of a molecule or the band structure of a material and its interband states such as surface and defect states. Applying optical spectroscopy to nanointerfaces is a great source of information. However, IR and UV/visible spectroscopy are bulk-sensitive (information length in the range of micrometers or millimeters) by default, and one must apply the aforementioned strategies to gather information from the interface.

In the case of noble metal nanoparticles, localized surface plasmon resonance (LSPR) occurring in the visible range^36^ may allow a significant reduction of the information volume (strategy S2). Surface plasmons are coherent electronic modes that exist at the interface between two materials. The absorption of the plasmon depends on the size and shape of the nanoparticles, as well as its interaction with the environment. For instance, the quantum size effects in silver nanoparticles are dominated by interfacial interactions, and the surface plasmon resonance frequency is sensitive to the local medium dielectric constant.^37^ It can be exploited for sensing chemicals, gases, and biological analytes.^38,39^

Fourier transform infrared (FTIR) spectroscopy, or IR spectroscopy for short, has been applied extensively to the characterization of aqueous solutions. It is especially sensitive to the water H-bonding environments and benefits from relatively simple set-ups. IR spectroscopy performed in transmission or diffuse reflectance geometry has a probing and information depth of several millimeters. Attenuated Total Reflectance (ATR) uses an evanescent wave which only penetrates a few micrometers into the sample deposited on top. Nevertheless, in the context of nanointerfaces, this geometry can still be considered bulk-sensitive, as the information depth is much larger than the characteristic length of the objects of interest. Therefore, in all three measurement geometries, the volume of the nanointerface has to be increased in order to match the volume probed with IR spectroscopy (strategy S1).

IR spectroscopy can also, in some cases, be surface-sensitive by reducing the information depth (strategy S2). For example, surface-enhanced infrared absorption spectroscopy (SEIRAS) is a technique that can achieve sensitivity down to single molecular layers in liquid.^40^ In SEIRAS, local surface plasmons created by metallic nanostructures enhance the electric field of the infrared light up to a factor of 10^5^,^41^ allowing the detection of as few as 500 molecules.^42^ SEIRAS has been used extensively in spectroelectrochemical studies of CO_2_ reduction^43,44^ and formic acid oxidation.^45,46^ Nevertheless, this technique is limited to adsorption on thin metallic films^43,44,46−49^ or, for best signal enhancement, very well-defined metallic nanostructures.^50−53^ It cannot be applied to the characterization of most nanointerfaces. A similar strategy can be applied to reduce the information volume of Raman spectroscopy, and we refer to recent reviews for more details on surface- and tip-enhanced Raman Spectroscopy (SERS/TERS).^54^

Finally, the use of interface-sensitive selection rules is another successful approach to probe nanointerfaces with optical spectroscopies (strategy S4). In particular, SFG and Second Harmonic generation (SHG) are based on the detection of nonlinear optical processes occurring due to the breaking of symmetry, such as at interfaces.^32,33^ These techniques have an extremely short probing depth. Theey have been mostly developed for flat interfaces, but have also been applied to probe some nanointerfaces, such as the graphene–water interface^55^ or buried perovskite layers.^56^ While developments in the characterization of colloidal nanoparticles have been made in recent years,^57^ the application of these techniques to nanointerfaces with a rough morphology in electrochemical cells is not straightforward at the current stage.

The use of surface selection rules at metallic substrates can also be applied to infrared spectroscopy. Infrared reflection–absorption spectroscopy (IRRAS) is based on the different intensities of p- and s-polarized light especially at grazing angles of incidence, allowing the detection of monolayers.^58^ By analyzing the reflection at different polarizations of the incident light, one can gain information on the orientation of adsorbed species.^59^ Also known as polarization modulation infrared reflection–absorption spectroscopy (PM-IRRAS), this extension of IRRAS overcomes the experimental difficulties caused by the lower reflectivity of water compared to a metal surface and allows the investigation of thin films on air–water interfaces.^60^ Again, IRRAS techniques are limited to smooth surfaces and are therefore not applicable to the characterization of nanoparticles, nanoporous materials, or layered nanosheets.

### 3.2 Infrared Spectroscopy

Many solid–water nanointerfaces have been investigated with FTIR while exposed to various environmental conditions such as heating, cooling, or changing humidity. In addition to probing interfacial water layers, IR spectroscopy is commonly used to monitor heterogeneous catalytic reactions in the gaseous phase.^61^ In this section, we highlight some examples of how different bulk-sensitive IR methods have been used to investigate nanointerfaces.

#### Transmission IR spectroscopy

Transmission IR spectroscopy is optically the simplest way to record IR spectra, since the infrared light goes through the sample at normal incidence. There is no need for additional optical components, and the absorption of light is independent of wavelength (unlike in ATR). However, this geometry places some restrictions on the sample. Powder samples must be pressed into a pellet, and the thickness of highly absorbing samples such as water must be controlled to avoid saturation of the absorption bands. Any investigations of nanointerfacial water must therefore be conducted in the absence of bulk water. Despite these limitations, transmission IR spectroscopy has proven to be a very useful technique to study interfacial and confined water in various materials such as nanotubes,^62,63^ MOFs,^64,65^ oxides,^66−68^ and biologically relevant systems.^69,70^

**Figure 5:**
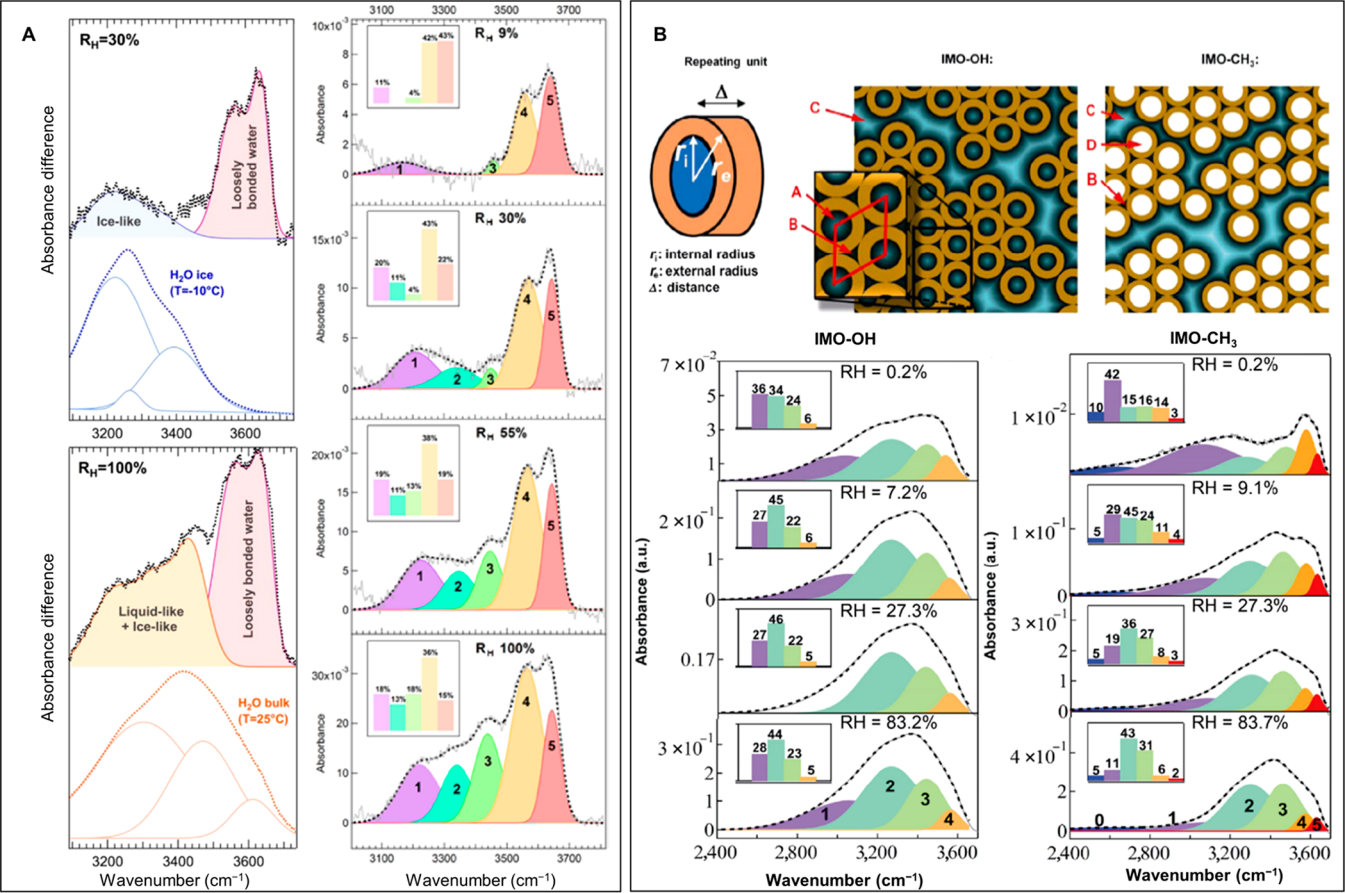
(a) IR absorbance water confined in CNTs at different relative humidities. A significant contribution from loosely bonded water was observed both in small diameter nanotubes, arising from a one-dimensional chain structure, and larger diameter nanotubes, arising from a water layer next to the nanotube wall. Adapted from ref (62). Copyright 2016 American Chemical Society. (b) Water confined in imogolite nanotubes with different surface chemistry. Water confinement was found to be governed by the hydrophilicity of the inner walls, and the interaction between water and the nanotube wall affected the degree of H-bonding between water molecules. Adapted with permission from ref (63). Copyright 2018 Springer Nature.

A fine control of the relative humidity over nanomaterials or nanostructured surfaces enables the investigation of interfacial water layers such as on carbon^62^ or imogolite^63^ nanotubes. At relatively high humidity, the nanotubes are filled with water, while no extensive liquid water film is formed due to the enhanced water condensation on nanotubes. In hydrophobic carbon nanotubes, even at fully hydrated state, dangling H-bonds dominate the IR spectrum (Figure 5). The water adsorption can further be controlled by the hydrophilicity of the nanotubes, leading to a wide variety of H-bonding as shown in Figure 5.

Confined water has been also investigated under varying temperature and pressure conditions, as confinement effects give rise to anomalous behavior and allow the study of water properties in the supercooled metastable state not accessible in bulk water.^71^ Water confined in MCM-41, a mesoporous silica containing cylindrical channels with a narrow pore size distribution, has been probed with transmission FTIR spectroscopy in the supercooled state.^71,72^ Interfacial water can be created by mixing small amounts of water with an organic phase that will cause the water to cluster into reverse micelle structures with nanoscale size. By ensuring that all water comes from confined environments of <5 nm micelles, Toda et al. were able to probe nanointerfacial water and observe a very distinct H-bonding signature compared to bulk-like water.^73^

#### Diffuse Reflectance Infrared Fourier Transform Spectroscopy

Diffuse reflectance infrared Fourier transform spectroscopy (DRIFTS) allows the direct investigation of powders without the requirement to apply pressure, as often is the case for transmission measurements using a KBr pellet, or ATR measurements where good contact with the ATR crystal is required. The benefits of this method are preserving the structure of the nanomaterial and better transport of reactants. DRIFTS is therefore particularly well adapted to nanoparticle-gas nanointerfaces and it has been used extensively in catalysis as evidenced by numerous recent reviews in the field focusing on carbon dioxide methanation on MOFs,^74^ metal–oxide-supported single atom catalysts^75^ and graphene-based materials for catalysis.^76^

**Figure 6:**
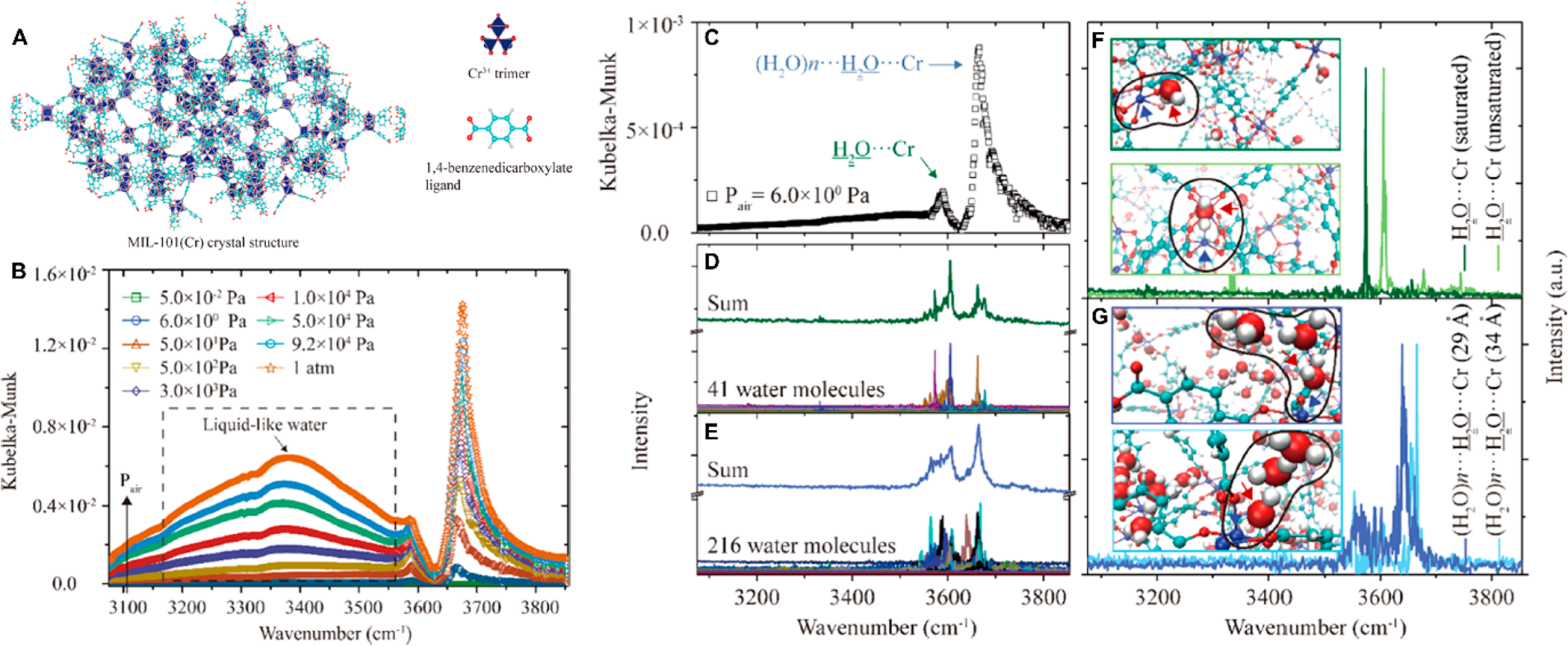
DRIFTS investigation of a MOF for heat-exchange applications. 1-dimensional water chains were identified at low pressure and bulk-like water filling the pores at high pressure. (A) Illustrations of the crystal structure of the MOF, the Cr^3+^ trimer, and the organic ligand. (B) DRIFTS spectra of the MOF at different air pressures. (C) DRIFTS spectrum of H_2_O···Cr and (H_2_O)*n*···H_2_O···Cr in the MIL-101(Cr) at 6.0 × 10^2^ Pa (*P*_air_). MD simulated vibration spectra for (D) the 41 water molecules (lower figure, each color line represents one water molecule) as well as the sum of all of the spectra (upper figure, in green) and (E) the 216 water molecules (lower figure, each color line represents one water molecule) as well as the sum of all of the spectra (upper figure, in blue). MD simulated vibration spectra of (F) single water molecule coordinated with the saturated (dark color) and unsaturated Cr^3+^ sites (light color), and (G) the first water molecule in the water chains for the 29 Å (dark color) and 34 Å cages (light color). Adapted from ref (77). Copyright 2021 American Chemical Society.

DRIFTS has also been utilized to study the interaction of water with MOFs.^77,78^ The chromium terephthalate-based MIL-101-Cr MOF was investigated at different pressures and the experimental spectra were compared to MD simulations (Figure 6). One-dimensional water chains coordinating with the Cr^3+^ centers at low pressure gradually grew into a monolayer of water on the inner surface of the MOF cages and changed the surface from hydrophobic to hydrophilic. This change in hydrophilicity induced water condensation at higher pressure, leading to the entire pores gradually filling with water as the pressure reached 1 atm.

#### Attenuated Total Reflectance IR spectroscopy

In the ATR mode, the infrared light is reflected internally off a prism made from a material with a high refractive index, such as Si or Ge. An evanescent wave is formed at the point of reflection that can be used to probe a sample in intimate contact with the prism. The benefit of the ATR mode is that the infrared beam does not pass through the sample, and therefore, the sample thickness does not need to be precisely controlled. This mode also enables various electrochemical geometries to be probed.^79−81^

**Figure 7:**
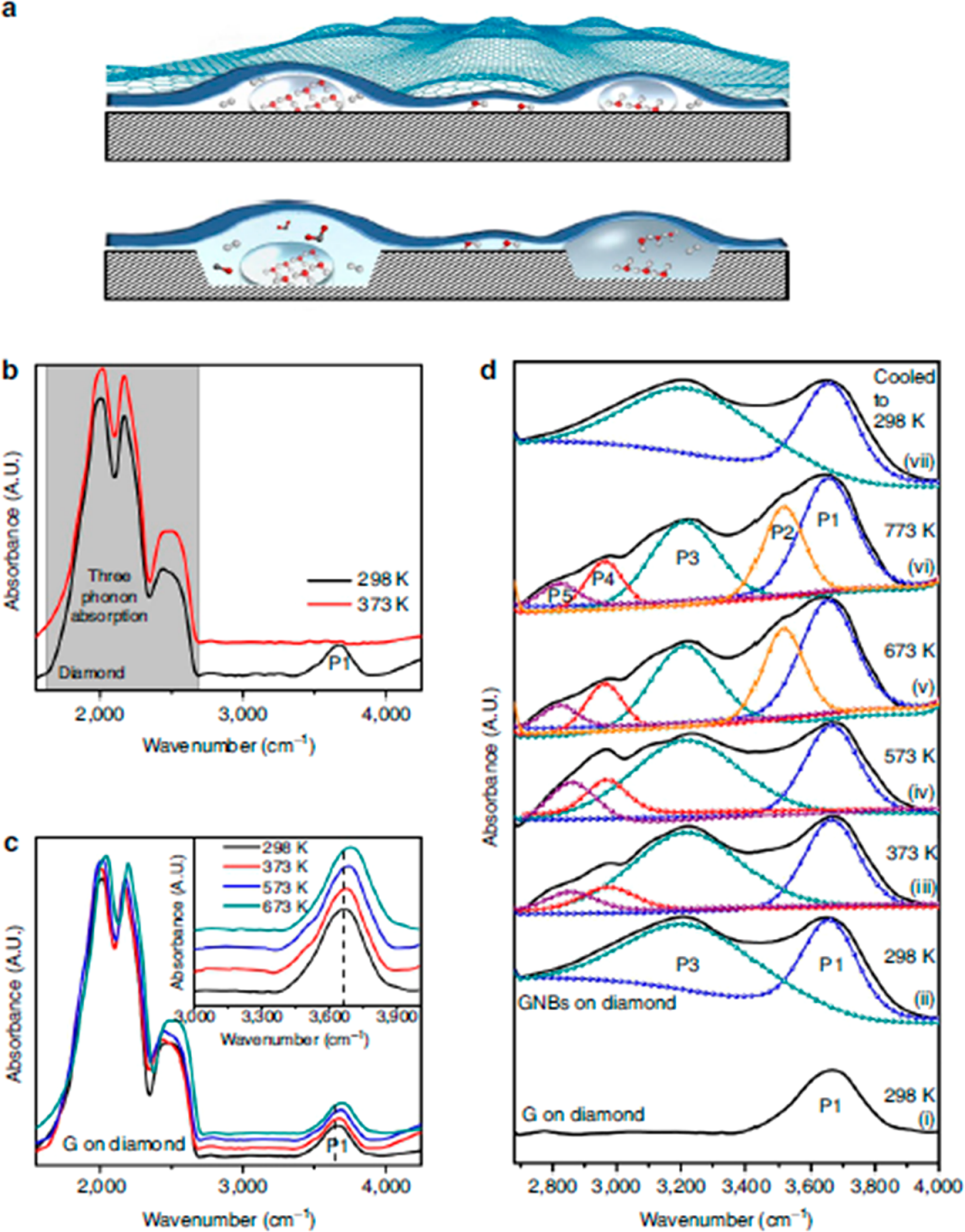
Nanoconfined water in bubbles between diamond and graphene probed by ATR-FTIR. (a) Schematic representation showing water cluster in graphene nanobubble (GNB) and weakly interacting water molecules underneath flat graphene on diamond (top panel). Etching of diamond by supercritical water (bottom panel). FTIR spectra showing OH-stretching peak of water measured on (b) diamond, where raising temperature to 373 K results in the desorption of water, (c) flat graphene on diamond showing peak at 3650 cm^–1^ due to the presence of trapped, weakly bonded water molecules, and (d) (i) flat graphene on diamond, (ii–vi) sample after formation of GNBs on diamond and heating the GNB at a range of temperatures, and (vii) sampe after cooling down to room temperature. Reprinted with permission from ref (83). Copyright 2013 Springer Nature.

When using the ATR mode to probe nanointerfaces and confined water, two strategies are possible. First, one can remove the bulk phase altogether. This strategy was employed to investigate confined water in reverse nanomicelles^82^ and nanobubbles.^83^ Suzuki et al. observed a rate-dependence in the formation of ice in reverse nanomicelles, with amorphous ice forming at slow cooling rates and metastable cubic ice when cooled rapidly.^82^ At elevated temperatures, Lim et al. reported unusual behavior of the water H-bonding in nanobubbles attributed to the formation of supercritical water due to the high pressure resulting from the nanoconfinement (Figure 7).^83^

Another strategy to transform ATR-FTIR into an interfacially sensitive technique is to reduce the bulk components of the nanointerface to be investigated up to a point that most of the probed volume is filled with interfacial phases. This is achieved by bringing a thick nanoparticle film in intimate contact with the ATR crystal. Using this strategy, the potential-induced protonation and deprotonation of electrolyte species and surface groups at the interface of a graphene nanoflake electrode was observed.^79^ In addition to water, the interaction of ionic liquids with nanoparticles is of interest for electrochemical energy storage applications. Richey et al. have conducted *operando* investigations using ATR-FTIR to elucidate how charge is stored in nanoporous carbon electrodes.^84,85^

**Figure 8:**
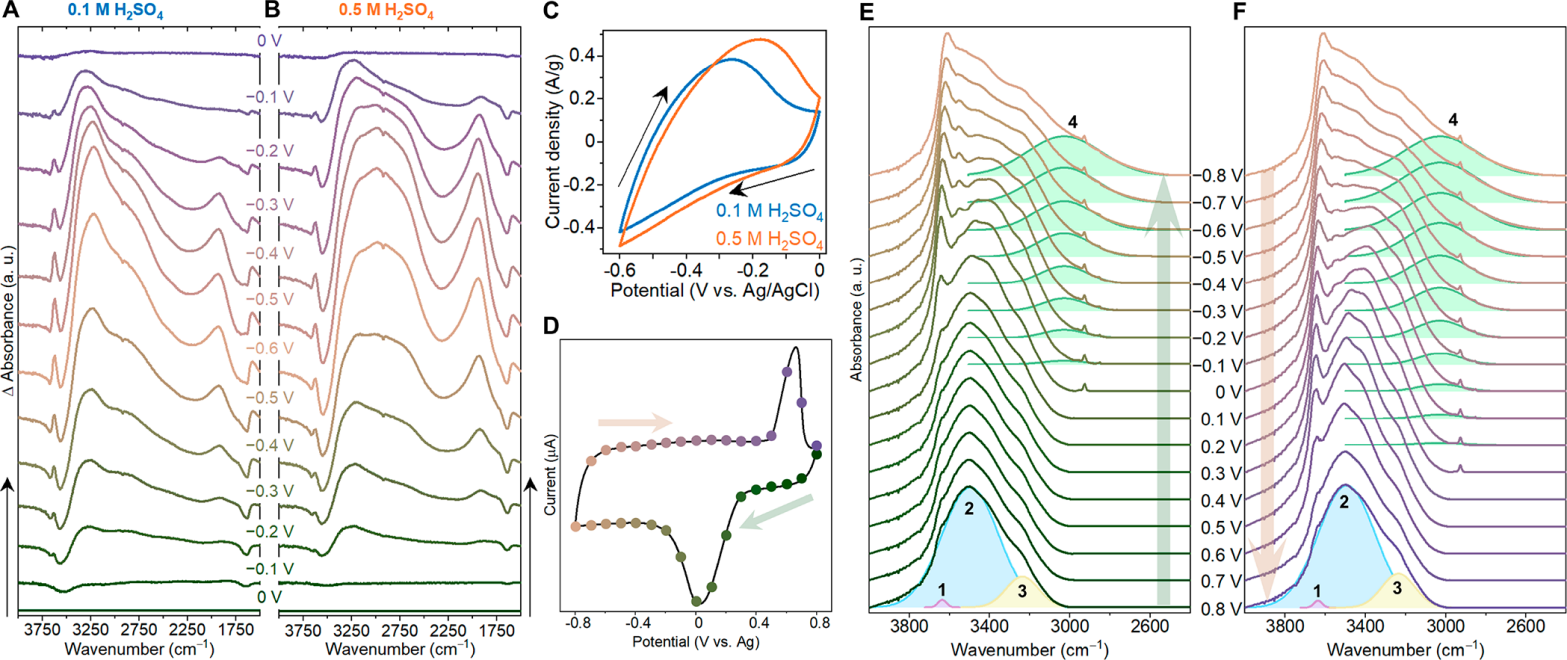
Nanoconfined water and protons in the nanoslits of layered Ti_3_C_2_ MXene. (A–C) Operando FTIR measurements of intercalated protons during electrochemical cycling in dilute sulfuric acid electrolyte. (D–F) Operando FTIR measurements of intercalated water during electrochemical cycling in highly concentrated LiCl electrolyte. Panels a–c adapted from ref (87). Copyright 2023 Springer Nature CC-BY 40. Panels d–f adapted from ref (88). Copyright 2023 American Chemical Society CC-BY 40.

Last but not least, nanolayered materials such as multilayered graphene oxide^67,86^ or MXene^87−89^ enable the investigation of confined water in the interlayer spaces. We recently employed this technique to probe protons and water intercalated in hydrophilic Ti_3_C_2_T_*x*_ MXenes (Figure 8).^87,88^ The thickness of the multilayered film deposited directly onto the ATR crystal enables a filtering of the bulk electrolyte component which is situated above the MXene film and hence only confined electrolyte can be probed. The possibility to apply potential is particularly interesting to probe various species in confined environment, which cannot be easily done with nanoconfined water in closed environment such as micelles or nanobubbles. During electrochemical cycling in dilute acidic electrolyte, discrete vibrational modes related to protons intercalated in the 2D slits between Ti_3_C_2_T_*x*_ MXene layers were detected.^87^ DFT calculations indicate that the hydrated protons have a lower coordination number in confinement, leading to a substantially different vibrational signature compared to the bulk case. In a LiCl water-in-salt electrolyte (Figure 8),^88^ the vibrational signature of the intercalated water was found to change significantly as a function of potential and closely correlating with the charging mechanisms observed in concentrated Li-based electrolytes.^90^

## 4 X-ray Spectroscopy of Nanointerfaces

### 4.1 Probing and Information Depths of X-ray Spectroscopies

X-ray spectroscopies are particularly relevant for nanointerfaces because they enable selective probing of the phase A or B when both phases consist of different elements (strategy S3), which is not possible with optical spectroscopies. X-ray spectroscopy excites core electrons of the atoms to unoccupied surface states or to vacuum, providing element specific information such as the oxidation state of the atoms or the chemical bonds it is involved with. They also offer a wide variety of detection techniques, which can provide information on different electronic states but can also be used to modulate the information depth. For XPS, based on the analysis of the kinetic energy of photoelectrons, the information depth can be tuned in the range of ∼0.1–10 nm by changing the X-ray excitation energy. This information depth remains limited to a few nanometers due to the short attenuation length of electrons in matter^30^ and therefore fits very well with the characteristic lengths of nanomaterials.^6^ While the short electron mean free path is an advantage for surface-sensitive studies in vacuum, XPS at solid–liquid interfaces remains challenging because it cannot be applied to buried nanointerfaces. Significant advances were achieved in this field using near-ambient pressure XPS,^91,92^ or graphene layers as ultrathin electron-transparent membranes,^93^ but it remains limited to an information depth of a few nanometers.

In X-ray absorption spectroscopy (XAS), the X-ray excitation energy is scanned over an energy range enabling transitions to partial unoccupied electronic states of the element of interest. Thanks to the fine-tuning of incoming X-rays, electronic transitions corresponding to a particular element and bonding state can be selectively excited. The probing depth will vary with the elements to be probed because it depends strongly on the elemental X-ray cross-section and the X-ray energy.^31^ The soft X-ray region, enabling the probing of light elements^94^ and of valence shells of transition metals,^95^ is particularly interesting for nanointerfaces, with probing depth ranging from several micrometers for nonabsorbing phases to a few hundreds of nanometers for absorbing phases. The downside to such a short attenuation length is the need for the measurement to be carried out under vacuum, which implies complex experimental set-ups. With probing depths of several millimeters, hard X-rays are well adapted for *in situ*/*operando* characterization in real devices but require the removal of bulk components for characterizing nanointerfaces (strategy S1).

The true X-ray absorption can be detected in transmission, but the detection of secondary processes following X-ray absorption is also possible. The main detection modes are fluorescence yield and electron yield, while ion yield has also been proposed in the gas and liquid phase.^96^ All these techniques have different information volumes which are shown in Figure 4 for the case of a nanocolloidal interface. In the soft X-ray range, photon-out methods (transmission and fluorescence yield) offer an information depth of a few tens of nanometers while electron-out (electron and ion yield) only provide information from the first few nanometers. Note that other X-ray spectroscopy techniques such as X-ray emission spectroscopy and resonant inelastic X-ray scattering can provide further information on occupied electronic states.^97^ These spectroscopy methods, also based on X-ray photon-out detection, have similar probing and information depths as fluorescence yield XAS and will not be discussed further. In the following, we will illustrate with selected examples how the different XAS detection techniques can be applied to characterize nanointerfaces.

### 4.2 X-ray Absorption Spectroscopy

#### Transmission and Fluorescence Yield Detection

**Figure 9:**
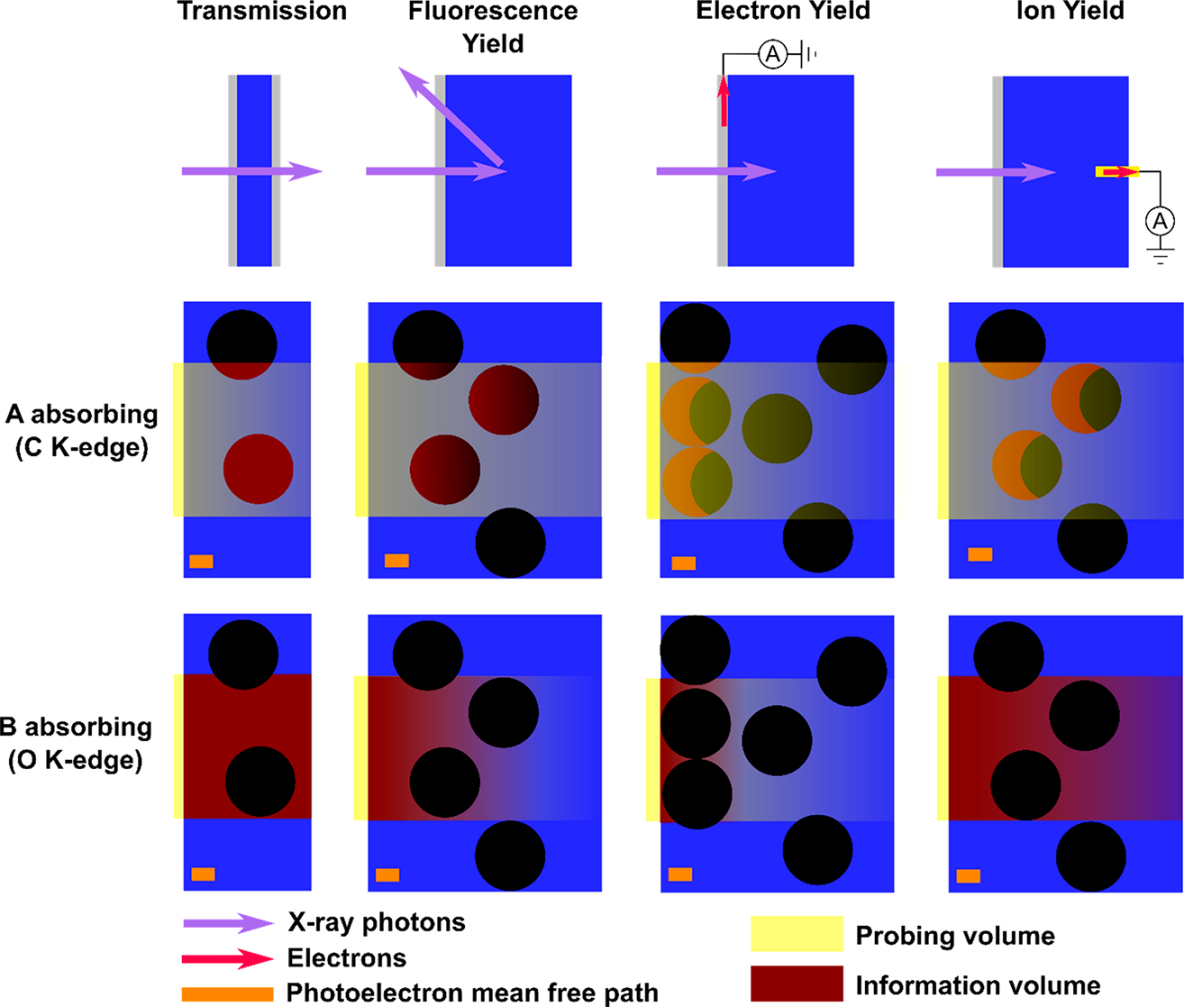
Schematic view of nanointerfaces probed by different XAS detection techniques. In this example, phase A (black) is a carbon-based nanoparticle and phase B (blue) an aqueous electrolyte. The element specificity is illustrated by showing two different excitation energies where A or B are absorbing the soft X-rays. The probing (yellow) and information (burgundy) volumes are indicated.

**Figure 10:**
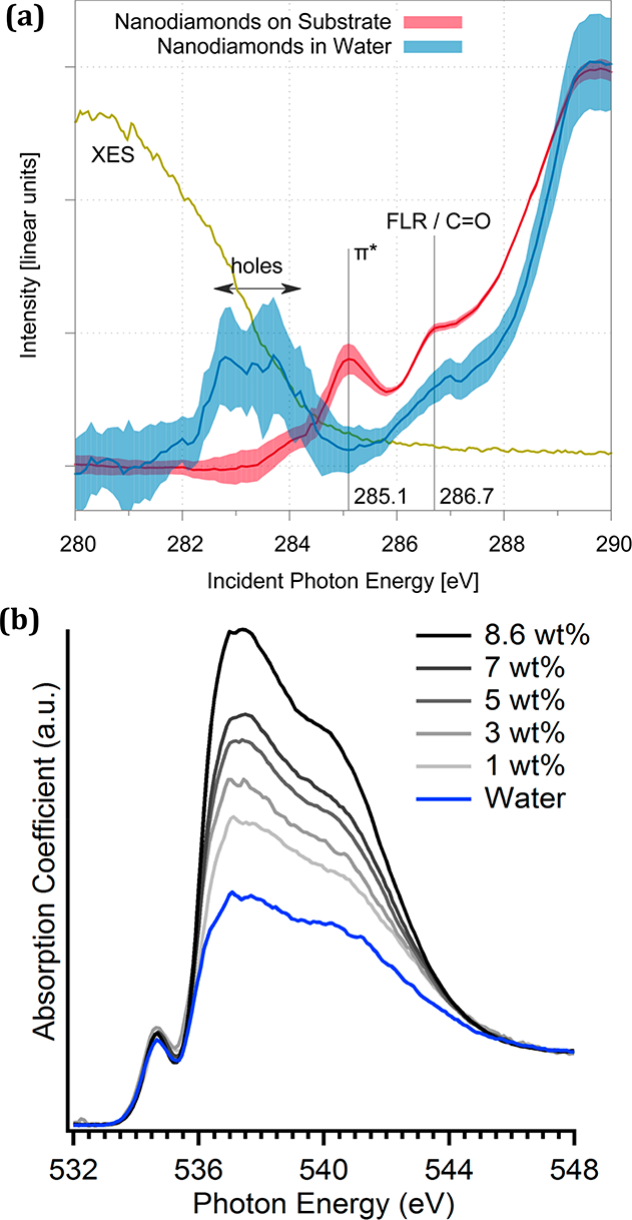
XAS of the nanodiamond-water interfacial region. XAS of nanodiamond aqueous dispersion at the carbon K-edge in fluorescence yield (a) and at the oxygen K-edge in transmission (b). Clear changes of the surface chemistry are observed at the carbon K-edge compared to dry nanodiamond while water reorganization is visible for increasing nanodiamond concentration at the oxygen K-edge. Panel a adapted with permission from ref (98). Copyright 2015 Royal Society of Chemistry. Panel b adapted with permission from ref (7). Copyright 2015 American Chemical Society.

In the context of solid–water nanointerfaces, the penetration depth in the range of 1 to 10 μm of X-rays in the so-called water window (280–535 eV) is a great asset.^31,94^ This ensures a significant probing volume for a nanophase with elements having absorption edges lying within the water window, such as carbon, nitrogen, titanium, or vanadium. Coupled with transmission and fluorescence yield detections, which have information depths of a few tens to hundreds of nanometers within absorbing materials, both the solid and the water phases can be selectively characterized by tuning the excitation energy as shown in Figure 9. This is typically the case for aqueous colloidal dispersions of carbon nanomaterials. Since the carbon K-edge (∼285 eV) lies in the water window, carbon atoms contained in the dispersed nanomaterials can be probed without large absorption of water molecules in the aqueous phase. The probing depth in pure water being in the order of 2 μm at the carbon K-edge, most of the X-ray absorption occurs in phase A because the carbon atoms are resonantly excited. Phase A will fully absorb the X-ray over a few tens of nanometers as depicted in Figure 9, which is on the order of magnitude of the characteristic length of nanoparticles. This enabled the observation of new unoccupied electronic states at the surface of nanodiamonds after dispersion in water using fluorescence yield detection (Figure 10a).^98^ Since nanodiamonds have a diameter (characteristic length) of ∼5 nm, the full volume of the nanodiamonds is probed by XAS. Due to their small size, most of atoms are contained within 1 nm from the surface, therefore XAS is still mostly sensitive to the interfacial region on such small nanoparticles. A similar approach was used to probe TiO_2_ nanoparticles in colloidal dispersions.^99,100^

Fluorescence detection is well adapted to probing dilute species but suffers from saturation effects when applied to concentrated phases due to self-absorption of X-ray photons,^101^ such as the aqueous phase in a nanocolloidal dispersion.^98^ As a result, the information volume of fluorescence yield is smaller than the probing volume and the spectra may be distorted for concentrated phases. On the other hand, transmission detection enables the measurement of absolute absorption cross sections, assuming that the sample can be made thin and homogeneous enough to allow reliable X-ray transmission.^102^ We applied previously transmission XAS to probe interfacial restructuring of water around nanodiamonds.^7,103^ In particular, a different H-bonding network was observed for increasing nanodiamond concentration (Figure 10),^7^ which was later found to be related to hydrogenated groups on the surface of nanodiamonds.^103^

**Figure 11:**
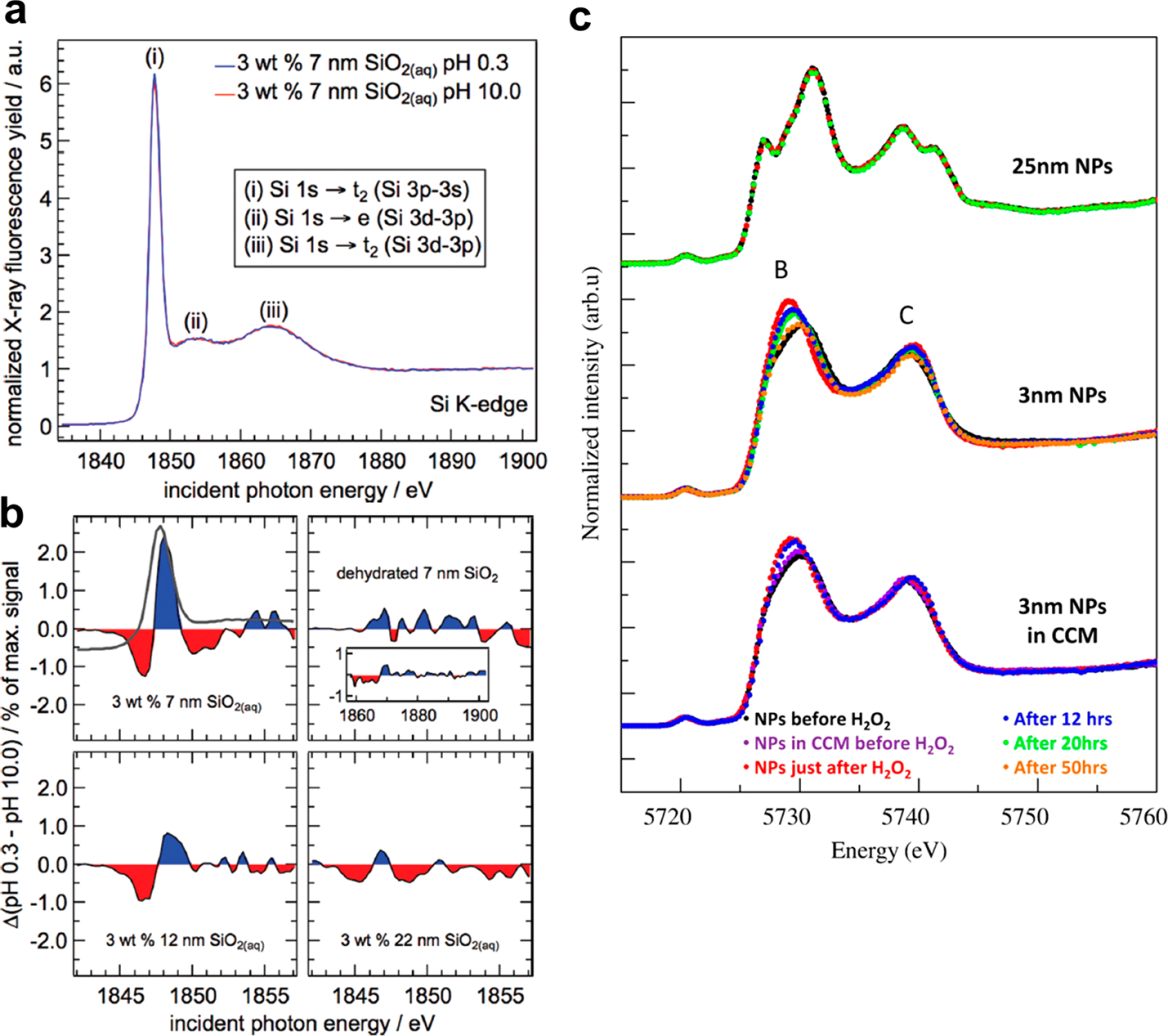
Interfacial reactions monitored on nanoparticle dispersions by XAS in the hard X-ray range. (a, b) Protonation of SiO_2_ nanoparticles is evidenced at the Si K-edge (a) and difference spectra between pH 0.3 and 10 (b) are found to depend on the nanoparticle size. (c) The Ce L3 XAS of ceria nanoparticles in water and cell culture medium (CCM) during the decomposition of H_2_O_2_ are shown. Panels a and b adapted from ref (104). Copyright 2012 American Chemical Society. Panel c adapted from ref (105). Copyright 2013 American Chemical Society.

When hard X-rays are required, both transmission and fluorescence yield can be easily performed because the X-ray penetration depth is much longer (few mm to cm). Despite the large probing volume, interfacial information can still be obtained when probing small colloidal nanoparticles (<10 nm) because of the high ratio of surface atoms to core atoms. An example is shown in Figure 11a,b, where the protonation of SiO_2_ nanoparticles with different sizes is measured at the Si K-edge.^104^ Spectral differences between extreme pH values are more visible on 7 nm nanoparticles than larger ones owing to the larger proportion of surface atoms. The monitoring of catalytic processes at the interface between water and ceria nanoparticles was also achieved on 3 nm nanoparticles due to the large interfacial component related to the small nanoparticles (Figure 11c).^105^

#### Electron and Ion Yield Detection

**Figure 12:**
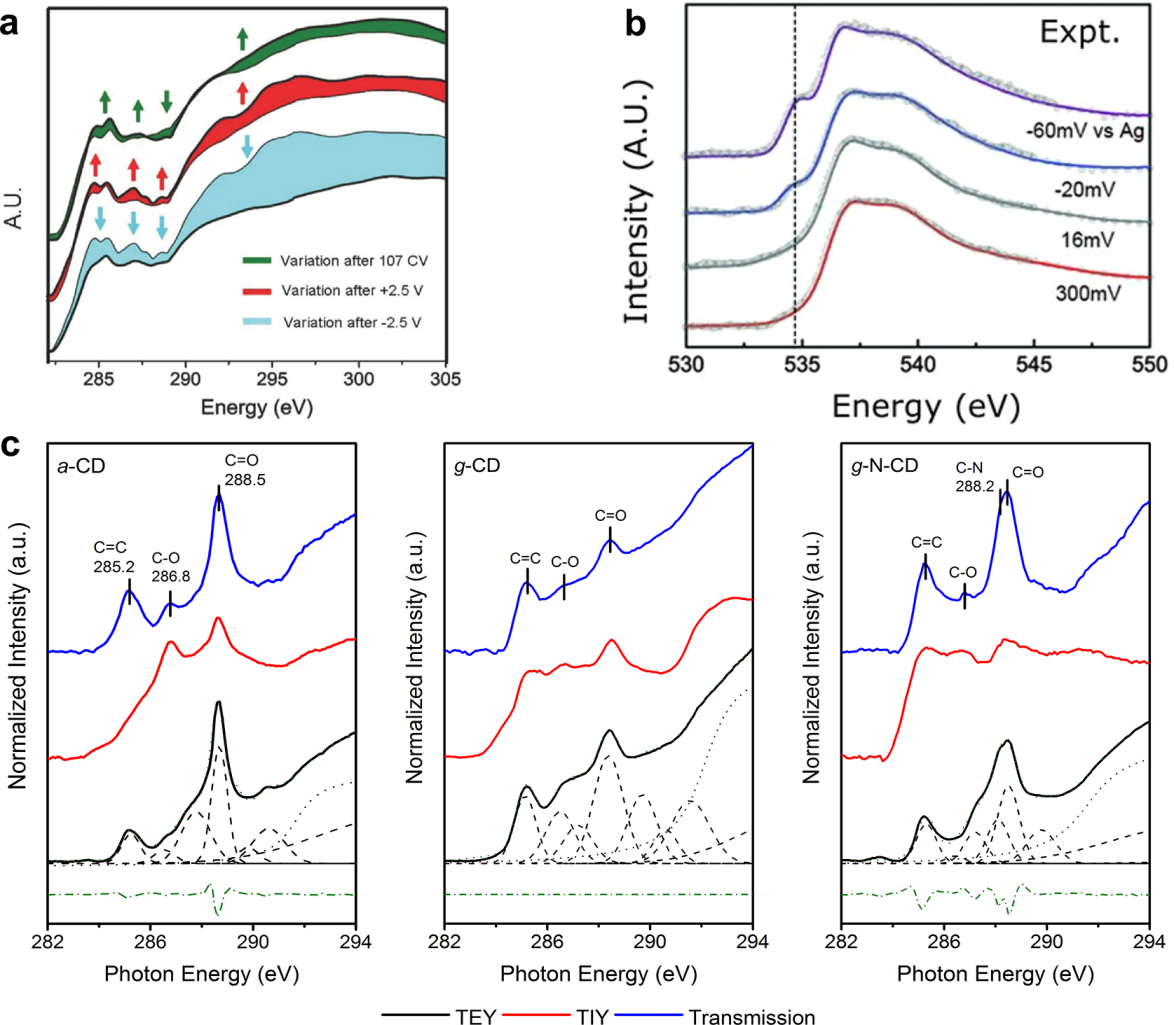
Electron and ionic yield XAS at solid–water interface. (a) Electron yield XAS at the carbon K-edge of graphene in water before and after applied potential. (b) Electron yield XAS at the O K-edge of water on a gold film at different applied potentials. (c) Comparison of XAS measured in different detection modes at the C K-edge of carbon dots with amorphous (aCD), graphitic (gCD) and nitrogen-doped graphitic cores (N-gCD) in water. Panel a adapted from ref (106). Copyright 2013 IOP Publishing. Panels b adapted from ref (107). Copyright 2014 American Association for the Advancement of Science. Panel c adapted from ref (109). Copyright 2019 American Chemical Society.

The short information depth of electron yield detection limited the characterization of samples in vacuum due to the low mean free path of photoelectrons as discussed earlier. Nevertheless, by measuring the drain current through a conductive sample exposed to a liquid, Velasco-Velez et al. showed the possibility to detect electron yield directly at solid–liquid interface.^106,107^ The advantage of this technique compared to XPS is that only secondary processes leading to a photocurrent are detected. As a result, the photoelectrons do not have to be detected directly and the current can be measured in the electrochemical cell using a simple ammeter. On the other hand, the origin of the detected photocurrent is still subject to debate. This process was first demonstrated on a graphene layer exposed to an applied potential and then characterized by XAS (Figure 12a).^106^ By introducing a lock-in detection, this technique was also used to characterize solvent restructuring at gold- and platinum-water interfaces under applied potential.^107,108^ Interestingly, the pre-edge observed at the oxygen K-edge of water was found to be highly sensitive to the applied potential (Figure 12b). This result was interpreted as a consequence of water reorientation in the first water layers at the solid–water interface, suggesting that the signal obtained through X-ray induced photocurrent has a very short information depth similar to electron yield in vacuum (a few nanometers).

Using a similar philosophy, Schön et al. showed that by measuring X-ray induced ionic current in solution instead of photocurrent flowing through a material in electrical contact with an electrode, XAS in solution can be measured.^110^ This detection scheme, called ion yield detection, relies on secondary processes resulting from photoelectron emission in liquid and was found to have an information depth closer to fluorescence yield than electron yield.^111^ In fact, since the ion yield detection relies on the diffusion of ions to the counter electrode, it does not suffer from the self-absorption of emitted X-ray photons and the information volume for concentrated species such as an electrolyte is larger than for fluorescence yield (Figure 9). We later used this detection scheme to probe charge transfer processes at solid–liquid nanointerfaces in aqueous dispersion of carbon dots.^109^ X-ray excitation of π*_C=C_ transitions, which led to X-ray absorption for transmission detection, did not induce an ionic yield signal for amorphous carbon dots unlike for graphitic carbon dots (Figure 12c). As a result, it was concluded that an efficient charge separation at the carbon dot–water interface is required for inducing an ionic current.

We have attempted to describe two different methods of detecting X-ray induced photocurrent signals as depicted in Figure 9. While electron yield is well suited to detect nanomaterials deposited on an X-ray transparent membrane with electrical contact, ion yield can provide information from dispersed nanomaterials in liquid. Note that the interplay between electron and ionic yield detection in liquid is still subject to discussion^112^ and requires further research. The development of X-ray induced photocurrent techniques, especially to charged nanointerfaces, will certainly contribute to a better understanding of electrochemical processes in the future.

## 5 Conclusions

Molecular processes at nanointerfaces are playing a major role in many applications and require adapted spectroscopic tools to probe them. We proposed here a definition and a classification of nanointerfaces, illustrated with solid–liquid nanointerfaces. We showed that the comparison of the characteristic lengths of the nanointerface of interest with the probing and information depth/volume of the spectroscopic technique is necessary to properly assess the volume which can be accessed. These considerations are particularly important when assessing the possibility to perform *in situ*/*operando* characterization of photo/electrochemical processes at nanointerfaces. Three further aspects were not discussed in this Perspective but are also essential when designing an experiment focusing on nanointerfaces:

- The temporal resolution of the spectroscopy technique is a critical parameter that Mmust be taken into account. Molecular processes occurring in the EDL at ultrashort time scales will not differ between classical and nanointerfaces. However, diffusion processes are likely to be much faster due to the reduced volume of nanointerfaces.
- The spatial resolution of the spectroscopy technique may be particularly important for some nanointerfaces. We assumed here that the nanointerfaces to be probed are homogeneously distributed through a large volume. However, this is likely not the case for many nanointerfaces, due to local inhomogeneities that may lead to different interfacial processes. In this case, nanospectroscopy techniques, enabling spatial resolutions of the same order of magnitude than single nanomaterials to be characterized, will be required.^54^
- Multimodal spectroscopy, combining different spectroscopy techniques measured simultaneously, is a promising approach that may offer complementary information from the different phases of a nanointerface. This can be achieved using correlation analysis between techniques with different selection rules^113^ and/or different information depths.^114^

While this Perspective was focused on experimental approaches, the coupling with theoretical calculations is often crucial to draw a full picture of chemical reactions at nanointerfaces. Molecular dynamics can provide crucial information on solvation and interfacial processes at the nanoscale, which are not always easily accessible by spectroscopy techniques.^115^ In addition, computational spectroscopy is generally of great help for the interpretation of spectroscopic results and must be included in the experimental workflow whenever possible.^116^ To conclude, we would like to emphasize that explicitly mentioning the probing, information, and characteristic lengths in further studies on nanointerfaces would be highly beneficial to facilitate the understanding of the results, especially when introducing new spectroscopic methods.
